# SARS-CoV-2 Testing for Asymptomatic Patients with Cancer Prior and during Treatment: A Single Centre Experience

**DOI:** 10.3390/curroncol28010032

**Published:** 2021-01-06

**Authors:** Nicholas Meti, Houman Tahmasebi, Angela Leahey, Angela Boudreau, Alia Thawer, Janice Stewart, Paige Reason, Kirsty Albright, Jerome A. Leis, Kevin Katz, Matthew C. Cheung, Simron Singh

**Affiliations:** 1 Faculty of Medicine, University of Toronto, Toronto, ON M5S 1A8, Canada; nick.meti@mail.utoronto.ca (N.M.); houman.tahmasebi@mail.utoronto.ca (H.T.); Jerome.Leis@Sunnybrook.ca (J.A.L.); Kevin.Katz@nygh.on.ca (K.K.); Matthew.Cheung@sunnybrook.ca (M.C.C.); 2 Division of Medical Oncology and Hematology, Department of Medicine, Sunnybrook Health Sciences Centre, Toronto, ON M4N 3M5, Canada; Angela.Leahey@sunnybrook.ca (A.L.); Angela.Boudreau@sunnybrook.ca (A.B.); janice.stewart@sunnybrook.ca (J.S.); paige.reason@sunnybrook.ca (P.R.); kirsty.albright@sunnybrook.ca (K.A.); 3 Department of Pharmacy, Sunnybrook Health Sciences Centre, Toronto, ON M4N 3M5, Canada; alia.thawer@sunnybrook.ca; 4 Division of Infectious Diseases, Department of Medicine, Sunnybrook Health Sciences Centre, Toronto, ON M4N 3M5, Canada; 5 Department of Laboratory Medicine, North York General Hospital, Toronto, ON M2K 1E1, Canada

## Abstract

Patients with cancer are more vulnerable to severe COVID-19. As a result, routine SARS-CoV-2 testing of asymptomatic patients with cancer is recommended prior to treatment. However, there is limited evidence of its clinical usefulness. The objective of this study is to evaluate the value of routine testing of asymptomatic patients with cancer. Asymptomatic patients with cancer attending Odette Cancer Centre (Toronto, ON, Canada) were tested for SARS-CoV-2 prior to and during treatment cycles. Results were compared to positivity rates of SARS-CoV-2 locally and provincially. All 890 asymptomatic patients tested negative. Positivity rates in the province were 1.5%, in hospital were 1.0%, and among OCC’s symptomatic cancer patients were 0% over the study period. Given our findings and the low SARS-CoV-2 community positivity rates, we recommend a dynamic testing model of asymptomatic patients that triggers testing during increasing community positivity rates of SARS-CoV-2.

## 1. Introduction

Patients with cancer are at an increased risk of developing severe coronavirus disease 2019 (COVID-19), caused by the severe acute respiratory syndrome coronavirus 2 (SARS-CoV-2) [1]. Early reports from Wuhan, China demonstrated that COVID-19 positive patients with cancer had an increased case fatality rate [2]. Worse clinical outcomes have been associated with recent exposure to chemotherapy [3]. These findings raise concerns about balancing the elevated risks of severe infection against delaying cancer treatment [4]. To reduce the risk of COVID-19 infection in patients with cancer, centers across the globe are reorganizing care delivery [5]. Although efforts to reduce exposure to SARS-CoV-2 will work to mitigate short-term spread, epidemiologic models suggest this pandemic will last months to years, highlighting the need for long-term solutions [6]. 

One strategy to prevent the development of COVID-19 in patients with cancer is to perform a naso-pharyngeal (NP) swab on all patients to detect SARS-CoV-2 prior to systemic therapy, regardless of symptoms. Although COVID-19 testing among asymptomatic cancer patients has been studied [7] and recommended by European Society of Medical Oncology guidelines [8], there is no prospective evidence that such testing of cancer patients is clinically meaningful or cost-effective. Considering the high cost and labor associated with this strategy, there is ambiguity whether this strategy will be clinically useful, especially with variable COVID-19 prevalence in different parts of the world and over different time periods within the pandemic itself given. The aim of this study is to assess the positivity rate of routine asymptomatic SARS-CoV-2 testing of patients with cancer prior to and during active anti-cancer therapy in the context of local and provincial positivity rates to better understand the usefulness of such a strategy during the COVID-19 pandemic.

## 2. Methods

This study was conducted at Odette Cancer Centre (OCC), part of Sunnybrook Health Sciences Centre (SHSC) in Toronto, Canada. All patients were screened for symptoms of COVID-19 prior to entry into OCC. Asymptomatic patients with cancer were prospectively tested for SARS-CoV-2 in two cohorts. Cohort A and B included patients who were set to start new anti-cancer therapy (i.e., systemic and/or radiation) and those already undergoing systemic treatments, respectively. Testing was done from 11 May to 3 July, 2020 for Cohort A and 15 June to 26 June, 2020 for Cohort B. 

Testing was performed on NP samples using Promega (Madison, Wisconsin) magnetic bead extraction kits and RT-PCR using primers targeting both the 5’UTR and E genes. Samples positive for both genes were considered positive. The Research Ethics Board of Sunnybrook Health Sciences Centre (Project Identification Number 2699) approved the study (informed consent forms were not required). 

As a concurrent measure of prevalence during the study period, we monitored COVID-19 positivity rate among symptomatic patients across our cancer center, our hospital COVID-19 assessment center, and the province of Ontario. Data obtained from Cohorts A and B were analyzed in the context of local and provincial COVID-19 positivity rate using hospital and publicly available testing data.

## 3. Results

A total of 300 patients were recruited to cohort A and 590 patients recruited to cohort B. In cohort A, the median age was 68 years and 40% were female; in cohort B, the median age was 63 years and 62% were female. In cohort A, the majority of patients (66%) were pre-radiation therapy; the majority of patients in cohort B were medical oncology patients undergoing systemic anti-cancer therapy, as summarized in Table 1. There were zero cases of COVID-19 positive in both cohort A and B. Provincial (Ontario) and hospital (SHSC) positivity rates over the study time period were 1.5% and 1.0%, respectively; testing of symptomatic (i.e., failed questionnaire screening) patients entering OCC was 0% over the same time period, as summarized in Table 2.

## 4. Discussion

Our study of 890 asymptomatic patients with cancer revealed no positive cases of COVID-19 in pre-treatment and during treatment clinical context. Although testing of all patients with cancer on active treatment for COVID-19 has been recommended [8], our results suggest that this practice might be an inefficient use of time and resources, such as staffing, testing reagents, and personal protective equipment (PPE), when test positivity rates of SARS-CoV-2 is low in the local environment. Similar to testing for influenza during peak seasons [9], when community positive testing rates of COVID-19 drop, widespread testing of patients should also decrease given the low likelihood of detecting a case. Based on these findings, we suggest evaluating the clinical and economic value of a dynamic model of testing that responds to test positivity rates of SARS-CoV-2 in the community as a more representative trigger of asymptomatic testing among cancer patients prior to and during active treatments. 

Inspired by models developed for the Centers for Disease Control in responding to multiple indicators of influenza activity [10], we recommend evaluating the benefit of using community SARS-CoV-2 test positivity rates to trigger asymptomatic testing among at-risk patient cancer patients. These triggers would be dependent on SARS-CoV-2 test positivity rates among symptomatic (i.e., that fail questionnaire screening at time of entry into the cancer center) cancer patients, local testing centers (e.g., hospital testing center), and the broader community (e.g., city-wide and/or province/state). Increasing positivity rates of any of these three indicators above pre-determined thresholds would result in re-introduction of asymptomatic testing at cancer centers to identify at-risk patients prior to anti-cancer therapy, as well as minimize the transmission of COVID-19 to other patients and staff. Our cancer center will be conducting a trial of using pre-set thresholds of cancer center, local testing center (i.e., hospital), and provincial SARS-CoV-2 positivity rates of 2%, 2%, and 5%, respectively, to trigger resumption of asymptomatic testing.

Limitations of our study include the fact that patients with cancer are generally well educated about infection risk mitigation and may represent a population of patients that have higher adherence rates to masking and hand sanitation, given the inherent infection risk of some anti-cancer therapies. In addition, our study time period was after lockdown measures across the city were enforced at the government level, which may have further limited the true community spread and risk of SARS-CoV-2 spread and resulted in lower prevalence of COVID-19. 

## 5. Conclusions

To our knowledge, this is the largest report of wide-spread testing among asymptomatic patients with cancer for SARS-CoV-2 prior to and during anti-cancer therapies. Understanding the value of such an approach will be essential in managing the COVID-19 pandemic as we enter second and subsequent waves. This will be especially relevant given the re-opening of our economy to ensure an agile approach to mitigating severe COVID-19 in patients with cancer. A dynamic model that responds to local and provincial SARS-CoV-2 positivity rate would mitigate against unintended treatment complications and SARS-CoV-2 transmission. 

## Figures and Tables

**Table 1:** Clinical characteristics of each cohort tested for SARS-CoV-2. * Concurrent chemoradiation.

Characteristics	Cohort A (*n* = 300)	Cohort B (*n* = 590)
**Patient Demographics**		
Median Age (Years; Range)	68 (27–97)	63 (26–92)
**Sex**		
Male	180 (60%)	222 (38%)
Female	120 (40%)	368 (62%)
**Patient Type**		
Medical Oncology	71 (24%)	516 (87%)
Hematology Oncology	30 (10%)	74 (12%)
Radiation Oncology	199 (66%)	16 (2.7%) *
**SARS-CoV-2 Test Result**		
Positive	0 (0%)	0 (0%)
Negative	300 (100%)	590 (100%)

**Table 2:** A summary of the positive testing rate at three different testing sites over the time period of testing cohorts A and B. OCC: Odette Cancer Centre; SHBC: Sunnybrook Health Sciences Centre. ^a^ This testing site was set up to test patients who failed screening at the entry point of the cancer center when asked if they had any symptoms of COVID-19. Numbers correspond to 11 May–3 July 2020. ^b^ Numbers were available weekly 10 May–4 July 2020, except for the week of 21–27 June (data not available).

Testing Characteristics	OCC Symptomatic ^a^	SHSC Test Centre ^b^	Ontario ^c^
No. Positive Tests	0	53	15,297
No. Tests performed	395	5112	1,047,903
Positivity Rate over study period	0%	1.0%	1.5%
Peak Positivity Rate (Date)	0% (N/A)	2.9% (May 17–23)	7.3% (May 19)
